# IL-2 as a therapeutic target for the restoration of Foxp3^+ ^regulatory T cell function in organ-specific autoimmunity: implications in pathophysiology and translation to human disease

**DOI:** 10.1186/1479-5876-8-113

**Published:** 2010-11-08

**Authors:** Eva d'Hennezel, Mara Kornete, Ciriaco A Piccirillo

**Affiliations:** 1 Department of Microbiology and Immunology, McGill University, 3775 University Street, Montreal, H3A 2B4, Qc, Quebec, Canada; 2 FOCIS Center of Excellence, Research Institute of the McGill University Health Center, 1650 Cedar Avenue, Montreal, H3G 1A4, Qc, Canada

## Abstract

Peripheral immune tolerance requires a finely controlled balance between tolerance to self-antigens and protective immunity against enteric and invading pathogens. Self-reactive T cells sometimes escape thymic clonal deletion, and can subsequently provoke autoimmune diseases such as type 1 diabetes (T1D) unless they are controlled by a network of tolerance mechanisms in the periphery, including CD4^+ ^regulatory T cells (T_reg_) cells. CD4^+ ^Treg cells are characterized by the constitutive expression of the IL-2Rα chain (CD25) and preferentially express the forkhead winged helix transcriptional regulator Foxp3. These cells have been shown to possess immunosuppressive properties towards various immune cell subsets and their defects are thought to contribute to many autoimmune disorders. Strong evidence shows that IL-2 is one of the important stimulatory signals for the development, function and fitness of Treg cells. The non-obese diabetic (NOD) mouse model, a prototypic model of spontaneous autoimmunity, mimics many features of human T1 D. Using this model, the contribution of the IL-2-IL-2R pathway to the development of T1 D and other autoimmune disorders has been extensively studied. In the past years, strong genetic and molecular evidence has indicated an essential role for the IL-2/IL-2R pathway in autoimmune disorders. Thus, the major role of IL-2 is to maintain immune tolerance by promoting Treg cell development, functional fitness and stability. Here we first summarize the genetic and experimental evidence demonstrating a role for IL-2 in autoimmunity, mainly through the study of the NOD mouse model, and analyze the cellular and molecular mechanisms of its action on Treg cells. We then move on to describe how this data can be translated to applications for human autoimmune diseases by using IL-2 as a therapeutic agent to restore Treg cell fitness, numbers and functions.

## Introduction

Peripheral immune tolerance requires a finely controlled balance between maintaining tolerance to self-antigens and mounting protective immunity against enteric and invading pathogens[1]. Self-reactive T cells sometimes escape thymic clonal deletion, and can subsequently provoke autoimmune diseases such as type 1 diabetes (T1D) unless they are controlled by a network of tolerance mechanisms in the periphery, including CD4^+ ^regulatory T cells (T_reg_) cells[2]. These cells constitute 1-10% of thymic and peripheral CD4^+ ^T cells in humans and mice, and arise during normal thymic lymphocyte development. T_reg _cells are characterized by the constitutive expression of the IL-2Rα chain (CD25) and preferentially express Foxp3, a forkhead winged helix transcriptional regulator, which is critical for their development and function[3]. CD4^+ ^T_reg _cells have been shown to possess immunosuppressive properties towards various immune cell subsets, although the mechanism varies according to the genetic background of the host, microflora and target tissue. As such, T_reg _depletion, or alterations of the *foxp3 *gene, as seen in *Scurfy *mice or IPEX patients, results in a loss of T_reg _cells, and catastrophic multi-organ autoimmunity[4,5]. Hence Treg cell homeostasis and function is necessary to the maintenance of peripheral tolerance, and their defect leads to organ-specific autoimmune disorders such as T1 D.

The non-obese diabetic (NOD) mice are a prototypic model of human autoimmunity as they spontaneously develop multi-organ autoimmune diseases including T1D[6]. T1 D is a chronic autoimmune disease that results in the destruction of the insulin-producing beta (β) cells of pancreatic islets of Langerhans, resulting in insulin deficiency and persistent hyperglycemia. Development of diabetes in NOD mice comprises several stages: a non-pathogenic peri-islet immune infiltration appears by 3-4 weeks (*checkpoint 1*). Following a clinically silent period, a progressive T cell-dependent destruction of the β islet cells occurs around 12 weeks of age (*checkpoint 2*)[7]. Coincident with the checkpoint 1 to 2 transition, a switch between regulatory and pro-inflammatory cytokine production occurs: prior to β cell death, a period of Th2-dominated (IL-4/IL-10), non-destructive insulitis is observed, followed by a destructive phase during which inflammatory cytokines such as IFNγ, TNF-α and IL-17 are produced. This step-wise progression, as well as cytokine switch, has led to the conclusion that waning immunoregulatory mechanisms were involved in T1 D pathogenesis[8-10]. Indeed, studies suggest that reduced CD4^+ ^T_reg _cell frequencies or function represent a primary predisposing factor to T1 D. Transfer of CD25-depleted splenocytes into NOD.*scid *hosts leads to a quicker onset of T1 D than total splenocytes[11]. A disruption of foxp3, B7/CD28 or CD40/CD40L pathways in NOD mice alters the thymic development and peripheral homeostasis of T_reg _cells, and leads to an accelerated T1 D onset compared to WT NOD mice[12,13]. Thus, T1 D onset/progression may be triggered by a reduction in Foxp3^+ ^T_reg _cell numbers and/or functions.

Strong evidence shows that IL-2, as well as other common gamma chain (γc; also known as CD132) signaling, are important stimulatory signals for the development, function and fitness of nTreg cells. Its signaling cascade is initiated by the binding of IL-2 to its trimeric IL-2 receptor (IL-2R) which consists of the α-chain (IL-2Rα; also known as CD25), the β-chain (IL-2Rβ; also known as CD122) and the γc chain. All three subunits contribute towards IL-2 binding, but only IL-2Rγ and the γc are required for signal transduction. The IL-2Rβ and the γc are also components of other cytokine receptors, expressed by many cell types and tissue, whereas the IL-2Rα expression is mostly restricted to activated T cells, and Treg cells[14].

In recent years, strong genetic and molecular evidence has shown that the IL-2/IL-2R pathway promotes Treg cells development and functional fitness, and functional variations of this pathway can promote susceptibility to autoimmunity. Here, we review these recent findings and explore the role of the Treg/IL-2 axis in the pathophysiology of organ-specific autoimmune disorders such as T1 D the functional potential of IL-2 and its possible implication as a therapeutic agent in the context of autoimmunity.

### Genetic evidence for a role of IL-2 in autoimmunity

The IL-2-IL-2R pathway plays an essential role in the development of T1 D and other autoimmune disorders in humans and mice. IL-2 is well-described to promote activated T cell proliferation, survival and differentiation[15]. However, mice deficient for IL-2, IL-2Rα (CD25) or IL-2Rβ (CD122) die prematurely from a severe, multi-organ, autoimmune and lymphoproliferative disorder[16]. Similarly, rare genetic disorders due to mutations of the *il2, cd25 *or *stat5a/b *genes lead to autoimmune syndromes[17-19], emphasizing the importance of IL-2 in the maintenance of self-tolerance[16].

### Il2 allelic variation (Idd3) and resistance to autoimmunity in NOD mice

T1 D susceptibility is inherited through multiple genes, with a strong predisposition for those affecting T cell responses to γ cells[7]. At present, genomic mapping studies of congenic NOD mice have identified 20 insulin-dependent regions (Idd) that influence either the onset insulitis, progression to overt T1 D, or both[20]. No single gene is both necessary and sufficient for T1 D susceptibility. Of particular interest is the Idd3 locus which was mapped to a 0.15-cM interval on the proximal mouse chromosome 3 between the microsatellite markers D3Nds55 and D3Nds40b[20-22]. Fine mapping studies show that the Idd3 locus encompasses several genes of potential immune relevance, notably: *Il-2*, testis nuclear RNA-binding protein (*Tenr*), *Il-21*, Centrin 4 (*Cetn4*) and Fibroblast growth factor 2 (*Fgf2*)[20], although the *IL-2 *gene is the strongest and primary candidate gene for protection in NOD mice congenic for the C57BL/6 *Idd3 *locus[20,22]. NOD mice introgressed with the protective Idd3 allele from C57BL/6 display a reduced onset and severity of T1 D, as well as reduced susceptibility to other organ-specific autoimmune disorders, such as experimental autoimmune encephalomyelitis (EAE) and autoimmune ovarian dysgenesis[23]. Yamanouchi *et al*. showed that expression of protective Idd3 alleles in CD8^+ ^T cells results in a 2-fold increase in IL-2 transcription and protein production compared to susceptible alleles[22]. The protection conferred by the Idd3 C57BL/6 allele can be explained by the presence of 46 SNPs upstream of the minimal promoter of the *IL-2 *gene that can alter the transcriptional activity of this gene compared to NOD mice[22]. Moreover, polymorphisms in *il2 *exon 1 cause multiple amino acid changes that have been proposed to be responsible for a differential glycosylation pattern[24]. As such, the presence of a proline rather than a serine at position 6 of the mature IL-2 protein, is associated with an increased glycosylation and prolongation of the IL-2 half life[24]. However, NOD.CZECH Idd3 mice, whose IL-2 glycosylation pattern is identical to that of wild-type NOD mice, is resistant to T1 D, suggesting that glycosylation differences, on their own, do not account for T1 D protection in NOD.B6 Idd3 mice[22]. Candidate-gene approaches have also demonstrated a role for the Idd3 locus in human celiac disease and RA[25], as well as in T1D[26,27]. Interestingly, neither the Idd3 locus, nor any of the candidate genes enclosed therein (*il-2, il-21*), have been identified as correlating with T1 D in the recent genome-wide association studies (GWAS).

### cd25 genetic polymorphisms are associated with human T1 D

Genetic evidence linking the IL-2/IL-2RA pathway to the predisposition of human autoimmunity, and in particular T1 D, has also emerged in recent years. First, Vella *et al*. observed that SNPs in the *cd25 *gene indeed correlate with T1 D in a large European cohort. However, this quite large interval also encompasses other immune-relevant genes such as IL15RA, and the authors could not pin-point the causal variant with the locus[28]. The genetic interval was significantly narrowed down thanks to the power of GWAS performed on large cohorts around the world. As such, two sets of SNPs have been identified in the 5' and 3' vicinity of the promoter of *IL-2RA*[29-31]. The molecular and functional consequences of these SNPs remain to be characterized, however they seemingly do not cause splicing variations, nor do they directly affect the five known promoter regulatory regions of CD25[31]. Some insights could come from the observation that levels of the soluble form of CD25 (sIL-2-RA) are slightly lower in the serum of patients carrying predisposing alleles[31], although the functional relevance of sIL-2-RA is ill-defined. Indeed, sIL-2RA seems to be able to partially block signaling downstream of IL-2 *in vitro*, all-the-while enhancing T cell activation and proliferation[32], a finding reminiscent of the recent observation on the impact of IL-2/anti-IL-2 mAb complexes (discussed below).

A study by Qu *et al*. observed an allelic imbalance of the CD25 SNP variants whereby the susceptibility haplotype correlates with lower CD25 mRNA in lymphoblastoid cell lines[33]. In accordance, it was simultaneously shown that CD4^+ ^T cells of the memory subset display higher surface expression levels of CD25 in patients harboring a predisposing allele[34]. CD25 SNPs have been suggested to affect the onset and progression to T1 D. Indeed, a study of late-onset T1 D in a Finnish cohort suggested that the predisposing SNPs originally described by Lowe *et al*. also correlate with the age of onset, and do so as strongly as the HLA-DQ2/DQ8 predisposing haplotype[35]. Furthermore, the predisposing haplotype of CD25 SNPs described by Qu *et al*.[29] was found to correlate with acute-onset diabetes, but not slow-onset or fulminant, in a Japanese cohort[36].

### Role of IL-2 in stabilizing Foxp3^+ ^Treg cells homeostasis in T1 D progression

#### Defective Treg cell fitness and survival in target organ as a trigger of autoimmunity

Several lines of evidence point to a critical role of the IL-2/IL-2R pathway in Treg cell development, function and homeostasis in human and murine autoimmunity. First, we and others have asked whether a possible quantitative or qualitative deficiency in Foxp3^+^CD4^+ ^Treg cells contributes to the onset and establishment of autoimmune diabetes in NOD mice[8]. We showed that thymic and peripheral CD4^+^CD25^+ ^T cells are fully functional *in vitro *and *in vivo *in both normal NOD mice and the BDC2.5 antigen-specific model of T1D[8]. Furthermore, Treg cells do not affect the priming or expansion of antigen-specific diabetogenic T cells in pancreatic lymph nodes, but regulate late events of diabetogenesis by localizing in the pancreas where they suppress the accumulation and function of effector Th1 and Th17 cells[8]. Interestingly, the function of Treg cells, while fully operative in neonatal mice, declines progressively with age[8]. The proportion of Foxp3^+ ^Treg cells in secondary lymphoid tissues is similar in the NOD mice relative to T1D-resistant C57BL/6 mice While T1 D progression is not attributed to systemic fluctuations in CD4^+^Foxp3^+ ^Treg cell numbers, there is a paradoxical increase of Treg cells in the pancLN at T1 D onset[8]. Interestingly, the transition from peri-insulitis (checkpoint 1) stage to T1 D onset (checkpoint2) is associated with a progressive loss of CD4^+^Foxp3^+ ^Treg cells in pancreas, but not in the pancLN, which in turn perturbs the Treg/Teff cell balance and allows the triggering of Teff cell pathogenicity in inflamed islets[8]. Moreover, intra-islet Treg cells expressed reduced amounts of CD25 and Bcl-2 relative to Treg cells in the pLN, suggesting that the Treg/Teff cell imbalance was due a defect in intra-islet Treg survival[10]. Collectively, these studies suggest that T1 D onset is associated with a loss of Treg cells numbers or/and function[37-42]. Several findings suggest that IL-2/IL-2R signaling is necessary for the peripheral maintenance and fitness of Treg cells. In Fontenot *et al*., the analysis of Foxp3-GFP reporter knock-in mice genetically deficient for IL-2 or IL-2R (CD25) revealed that IL-2 signaling is not required for the induction of Foxp3 expression in thymocytes. These findings were further confirmed by demonstrating that Treg cell development is independent of IL-2, while this cytokine is essential for survival of Treg cells[43]. Moreover, although IL-2^-/- ^or IL-2R^-/- ^mice display reduced numbers of Treg cells *in vivo*, their suppressive function *in vitro *remains unaffected[44]. Nonetheless, gene expression analysis showed that IL-2 signaling was required for the maintenance of the expression of the genes involved in the regulation of cell growth and metabolism[22]. Hence, IL-2 has a critical role in the homeostasis and competitive fitness of Treg cells[3]. Interestingly, the adoptive transfer of WT Treg cells either in IL-2^-/- ^or IL-2R^-/- ^mice can only prevent autoimmunity in IL-2R^-/-^, and not IL-2^-/-^, mice[16,45]. These results indicate that the lack of Treg cells in IL-2^-/- ^and IL-2R^-/- ^mice contributes to the autoimmune phenotype and that IL-2 maintains self tolerance by increasing the number of Treg cells present in the peripheral organs[46].

Similarly, T cell-specific deletion of STAT5a/b leads to reduced Treg cell numbers[47]. Antov *et al*. demonstrated that adoptive transfer of C57BL/6 background WT mice CD4^+^CD25^+ ^Treg cells into STAT5^-/-^, mice was sufficient to prevent the development of splenomegaly and autoimmunity, demonstrating that disease symptoms in STAT5 mice are due to defective Treg cells[48]. Another player in the IL-2 signaling cascades is the Jak3 kinase. Jak3^-/- ^mice display symptoms of autoimmunity and accumulation of auto-reactive T cells in the lymphoid organs[48]. It has been shown that the frequency of CD25^+^CD4^+ ^Treg cells in the spleen of Jak3^-/- ^mice was similar to that in IL-2^-/- ^and IL-2β^-/- ^mice, and was reduced compared to the C57BL/6 background WT mice[48]. Altogether, these findings indicate that Jak3 and STAT5a/b signals are required to maintain normal numbers of Treg cells in peripheral lymphoid organs and maintain self-tolerance downstream of IL-2/IL-2R signaling. Overall, IL-2 may not be absolutely required for the thymic generation of Treg cells but is a critical contributor of peripheral tolerance by maintaining a fit Treg cell pool.

#### IL-2 restores the Treg/Teff balance in T1 D

The importance of IL-2 in the maintenance of Treg cell homeostasis and suppression in T1 D has been suggested by IL-2 neutralization studies[49]. Administration of an IL-2-neutralizing antibody into neonatal NOD mice precipitated T1 D development by selectively depleting the Treg cell subset, reinforcing the importance of IL-2 in promoting Treg cell functions[49]. Similarly, a recent study by Tang *et al*. showed that CD4^+ ^Teff from islets of NOD mice were selectively impaired to produce IL-2, consistent with s report documenting the appearance of TCR hyporesponsive T cells coincident with the development of insulitis[10]. Conversely, low dose administration of IL-2 in pre-diabetic NOD mice restored CD25 expression and survival in intra-islet T_reg _cells, increase of the overall frequency of Foxp3^+^CD25^+ ^Treg cells in islets and led to T1 D prevention[50]. Overall, these results show that an IL-2 deficiency contributes to intra-islet T_reg _cell dysfunction and progressive loss of self-tolerance in the islets.

As discussed above, the increased transcriptional activity of protective Idd3 alleles translates into higher levels of IL-2 production by auto-reactive CD8^+ ^T cells in response to antigenic stimulation and, controls the size of the Treg cell pool in the pancreatic lymph nodes of NOD mice[10,22] These results show that *IL-2 *gene variation may affect the balance between islet-specific auto-reactive T cells and Foxp3^+ ^Treg cells, and consequently precipitate T1 D. In Sgouroudis *et al*., we asked if *Il2 *allelic variation potentiates Foxp3^+ ^T_reg _cell-mediated regulation of T1D[9]. NOD.*Idd3*^B6 ^mice show a markedly reduced incidence and delayed T1 D onset compared to control NOD mice. This resistance is associated with significantly reduced insulitis scores and frequencies of IFN-γ, TNF-α and IL-17 secreting autoreactive CD4^+ ^T cells, and correlates with increased IL-2 gene expression and protein production in antigen-activated CD4^+ ^T_eff _cells[9]. The *Idd3*^B6 ^allele favors the suppressive functions of T_reg _cells *in vitro*, and this increased T_reg _cell function, in contrast to controls, restrains the expansion and effector functions of CD4^+ ^T_eff _cells more efficiently *in vivo*[9]. Interestingly, T1 D resistance in *Idd3*^B6 ^mice correlates with the ability of protective *Il2 *allelic variants to promote the expansion of T_reg _cells directly within islets undergoing autoimmune attack[9,51]. Thus, T1D-protective IL2 allelic variants impinge the development of γ-islet autoimmunity by bolstering the IL-2 production of pathogenic CD4^+ ^Teff cells, and in turn, driving the functional homeostasis of CD4^+^Foxp3^+ ^T_reg _cells in the target organ.

### Treg lineage commitment and stability of Foxp3 expression

IL-2 is important in instructing Treg lineage commitment. Apart from thymic-derived Treg cells, induced Treg cells can acquire Foxp3 expression following T cell activation in the periphery, a process that is facilitated by IL-2[52]. For example, TGF-γ1 induction of Foxp3-expressing Treg cells *in vitro *is highly dependent on IL-2. Recent evidence also points to the functional plasticity of Foxp3^+ ^T_reg _cells in which Foxp3 expression and suppressive activity can be modulated in pre-committed Foxp3^+ ^Treg cells depending on the inflammatory milieu. This is evidenced by a recent study by Zhou *et al*. which points out that a loss of Foxp3 expression within T_reg _cells has been described as a critical event which can break self-tolerance and trigger autoimmunity[53]. The ensuing unstable Foxp3^+ ^Treg cells acquire a pathogenic phenotype, as reflected by the production of pathogenic cytokines such as IFN- γ and IL-17, and contribute to the onset of T1D[53]. These results suggest that an IL-2 functional deficiency in the target organ may disturb the positive feedback loop that controls Foxp3 stability, such that T_reg _cells convert to Teff cells with a high diabetic potential. Moreover, Komatsu *et al*. noted that Foxp3^+ ^cells with low CD25 expression lose more Foxp3 expression and become effector T cells, where cells with high CD25 expression are more resistant to such a conversion[54]. These findings have important implications for the role of Foxp3 in Treg cell lineage commitment, suggesting a role of IL-2 as a key player in Treg cell plasticity and heterogeneity. These studies also shape our thinking as some human trials have been initiated that use Treg cells-based immunotherapy.

#### Molecular basis underlying IL-2 mediated Treg cell homeostasis

Recent evidence shows that microRNAs (miRNA) can play an important role in the regulation of immunological responses by influencing Foxp3 stability[55-57]. As such, it has been shown that when DICER, a molecule critical to the function of miRNA, is deleted, Treg cells down-regulate Foxp3 expression, adopt an effector-like phenotype, and mice rapidly develop a fatal systemic autoimmune disease resembling the Scurfy syndrome[58]. More specifically, miRNA155 is preferentially expressed in Foxp3^+ ^cells, and a miR155 deficiency results in an increased suppressor of cytokine signaling 1 (SOCS1) activity in Treg cells, which has been previously described as a negative regulator of the IL-2 signaling. Furthermore, miR155-deficient Treg cells display a low proliferative capacity in response to limiting amounts of IL-2, whereas high amounts of IL-2 lead to normal levels of STAT5 phosphorylation[55]. Hence miRNA155 is required for Treg cell fitness in contexts of differential IL-2 levels in contexts of homeostasis and inflammation. Therefore, the waning of Treg cells, and ensuing breakdown in the self tolerance, could depend on the *in situ *IL-2 environment. These data all together suggest that Treg cell stability and their responsiveness to the IL-2 can be controlled by different miRNA therefore opening new avenues for potential therapeutic targets for the prevention and treatment of autoimmune disorders.

IL-2 may also directly impact the survival of Foxp3^+ ^Treg cells by promoting the expression of CD25 and Bcl2, a critical anti-apoptotic gene in T cells. Indeed, Tang *et al*. have shown that progression from peri-insulitis to destructive insulitis in the NOD mice correlates with intra-islet Treg cells expressing decreased levels of CD25 and Bcl2. These data suggest that Treg cells decrease in number by apoptosis due to a deficiency of IL-2 in inflammatory sites[10]. Hence, IL-2 may function as critical an anti-apoptotic factor for Treg cells.

#### Evidence of Treg deficiencies in human T1 D

It is unclear whether a quantitative or qualitative Treg cells defect contributes to human T1 D pathogenesis. Indeed, some studies claim a numerical defect[59], others a functional one[38,60], some none at all[61,62]. Defining Treg cells in human is much more challenging than in mouse due to the lack of stringency of FOXP3 expression as a marker of Treg cells. Indeed, in humans, FOXP3 is expressed by activated Teff cells[63], and forced or natural expression of FOXP3 does not always correlate with a regulatory function[2,64](our unpublished data).

The association between IL-2 and Treg cells in humans has also presented with more challenges than in murine work, due to the lack of reliable phenotypic markers discriminating human Treg from Teff cell populations. *In vitro *studies have shown the absolute necessity of IL-2 for the maintenance of FOXP3 expression and maintenance of the suppressive phenotype in Treg-enriched CD4^+^CD25^+ ^cells[65,66]. Accordingly, it was further shown that Treg-enriched CD4^+^CD25^+ ^cells isolated from diabetic subjects displayed a concomitant defect in IL-2 signaling and a difficulty to maintain FOXP3 expression levels even in the presence of IL-2[67]. This study does not, however, address whether a potential loss of suppressive function correlates with FOXP3 loss. Interestingly, the lack of suppression of auto-reactive T-cells from peripheral blood of subjects after the clinical onset of T1 D is due an increased apoptosis in Treg cells, possibly mediated by deprivation of growth signals such as IL-2[68]. Hence, IL-2 has a potential critical role in the fitness and/or lineage maintenance of human Treg cells, which is likely one of the major mechanisms by which the IL-2/IL-2-RA pathway impacts T1 D resistance in humans.

#### Autoantigen-driven Treg cell defects in organ-specific autoimmunity?

An important aspect in our understanding of the pathogenesis of autoimmunity is that potential immune defects may only be apparent when and if they affect autoantigen-specific fractions within Teff or Treg cell compartments. Indeed, the onset of organ-specific autoimmune disorders such as T1 D, MS and RA, can be interpreted in two ways: 1) a cell-autonomous, genetically-driven, defect exists in autoantigen-specific Treg cells, in turn leaving the activities of autoantigen-specific Teff unchecked in a given organ. The local inflammatory micro-environment or the degree of functional Treg ablation are contributing factors which may unveil this Treg defect, and in turn, mark the transition to overt autoimmunity; and 2) the autoantigen-specific Treg cell pool remain unaffected but genetic variation influences immune selection and/or activation of antigen-specific, pathogenic T cells, leading to a breakdown of self tolerance in a given organ. These two scenarios are of course non mutually-exclusive in individual subjects.

In the implications of such considerations lies the relevance of studies examining defects on a global population of Treg cells obtained from the peripheral blood, as opposed to examining the defects solely in the antigen-specific subset of T cells, and Treg cells in particular. Indeed, only islet-specific T cells can enter the pancreas to contribute to diabetes[69]. Additionally, the T cells found in the blood, whether it be in their repertoire, function and state of activation, may not accurately reflect the status and behavior of their counterparts localized in the target organ.

In this latter regard, there is experimental evidence that the blood carries at least a fraction of those cells with undeniable pathogenic potential. As such, it has been shown that beta islet cell-specific CD8^+ ^T cells can be found in the blood of mice, that constitute a predictive marker of onset[70-72]. Furthermore, the number of islet-specific CD4^+ ^T cells increases in the blood of pre-diabetic mice in correlation with increased infiltration of pancreas, however, their repertoire, unlike CD8^+ ^cells, was found to be more restricted in the islet than in the blood[73]. The authors also point out that when taken in blood, antigen-specific CD4^+ ^T cells are less pathogenic, whereby when adoptively transferred, recipients do not develop disease unless the cells were obtained from islets[74]. Thus, caution is required when interpreting functional data obtained from peripheral blood.

In humans, islet-specific T cells are found in the blood of normal subjects, but are slightly more prevalent in T1 D patients or at-risk subjects[75,76]. Interestingly, only in at risk and T1 D patients does this subset exhibit markers of prior activation, namely the memory marker CD45RO[77,78]. Given the extremely low abundance of T1 D autoantigen-specific cells in the blood, combined with the very low frequency of Treg cells, it has not been elucidated yet whether or not quantitative or qualitative defects in T1 D auto-Ag specific Treg cells can be detected in the blood. Thus, observations from the blood, if not mimic, at least reflect events ongoing at the specific site of inflammation. Whether or not those events that are translated into the blood encompass autoantigen-specific Treg cell defects remains to be determined.

### Modulation of the IL-2/IL-2R pathway for therapeutic purposes

Given the strong link between IL-2 and autoimmunity, it seems appealing to consider the use of IL-2 as a therapeutic tool for T1 D. However, this might prove quite challenging, as IL-2 is first and foremost a T cell growth factor, and as such, has strong proliferative effects on all T cells, including pathogenic CD4^+ ^and CD8^+ ^Teff cells. For the past decade, IL-2 has been used in the treatment of several diseases where the immune system necessitates strengthening of the activated T cell pool. As such, IL-2 is a frequent therapy in the treatment of solid tumors, mainly melanoma and renal cancer. In such cases, high doses of IL-2 are injected frequently leading to tumour regression in only about 10% of patients, and devastating side effects. While Teff cells were believed to be the primary target of treatment in treated patients, a 4-fold increase in suppressive CD4^+^CD25^+^FOXP3^+ ^cells was described although immune responses in patients for whom IL-2 treatment had worked were not analyzed[79]. Hence the main hurdle to human IL-2 immunotherapy for T1 D is to obtain an efficient and timely targeting of activated Treg versus Teff cells during distinct phases of T1 D progression. Several studies report the use of several strategies to modulate IL-2 signals and ultimately impact the Teff/Treg balance *in vivo*:

#### Low dose IL-2 prophylaxis therapy

Treg cells differ from their Teff counterparts in their IL-2 signaling pathways. Indeed, Treg cells are able to form the highest affinity receptor complex for IL-2, due to their constitutive expression of CD25, making them especially sensitive to very low levels of IL-2, in a fashion that seems to be relatively independent of the IL-2Rγ chain[80]. This supports the rationale of examining the potential of low-dose IL-2 as a "Treg-only enhancing treatment". Low-dose IL-2 has been used for several years to facilitate hematopoietic stem cell transplantation (HSCT). Studies in such patients indicate that Treg cells do increase in response to the treatment, and that this effect seems to be increased with prolonged time of treatment[66]. Of note is the fact that this effect correlates with a medically positive outcome, i.e. absence of graft rejection and GVHD. Accordingly, a low-dose IL-2 regimen diminishes the magnitude and frequency of CTL responses to a peptide vaccine against melanoma[81]. These observations are consistent with a recent report showing that administration of low-dose IL-2 promoted Treg cell survival and protected mice from developing diabetes in NOD mice[10].

#### Anti-IL-2 blockade in vivo

One explanation for the initially observed need for high doses of IL-2 in the treatment of cancer might have originated from the very short half-life of purified IL-2 after injection (3-5 min in mice)[82]. However, high-dose IL-2 leads to a devastating syndrome resembling septic shock. Hence, several avenues have been explored in order to stabilize the molecule *in vivo*, allowing for lower doses to reach sufficient therapeutic potency. As such, fusion with a carrier protein such as gelatin, BSA or even an irrelevant immunoglobulin chain have successfully prolonged IL-2 half life and reduced the side effects[82].

The undesired emergence of Treg cells has been pointed out as a potential culprit for treatment failure in cancer. Thus, focus has been put on modulating the affinity of IL-2 for its receptor complexes. Indeed, if IL-2 could be made to have a greater affinity for IL-2Rγ than IL-2Rα, the preferential bias of Treg cells in receiving IL-2 signaling would be cancelled out. As such, targeted mutations of the IL-2/IL-2RA binding sites have shown promising results[82].

More recently, a novel therapeutic tool has emerged that enables both higher stability, and selective cellular targeting of IL-2 *in vivo*. Indeed, binding of IL-2 to its receptor complexes could also be modulated by coupling IL-2 with different anti-IL-2 monoclonal antibodies (mAb). By varying the clone of the mAb, IL-2 can be targeted preferentially towards either CD25 or CD122[82,83]. These complexes, when "stimulating", show a therapeutic effect *in vivo *in mice[84]. However their exact mechanism of action remains unclear. Recently, it was shown that the effect of the stimulating IL-2/anti-IL-2 mAb complex treatment is recapitulated by a conjoint prolongation of IL-2 half-life and a blockade of CD25. Moreover, the effect of IL-2/anti-IL-2 mAb does not depend on FcRs[85].

#### Combination therapy with rapamycin

Another way of selectively targeting Treg cells could be the use of pharmacological agents that selectively modulate biochemical pathways in Teff or Treg cells. Rapamycin (Sirolimus) is a commonly used immunosuppressive drug which targets the cytosolic protein FK-binding protein 12 (FKBP12) and downstream mTOR pathway, and in turn inhibits IL-2 responsiveness in activated T cells[86]. Investigations into its mechanism of action have highlighted that Treg cells respond differently than Teff. Indeed, upon rapamycin treatment, Treg cells upregulate anti-apoptotic, and down-regulate pro-apoptotic molecules[87,88], in turn altering the Teff/Treg balance. Interestingly, the same anti-apoptotic molecules were increased downstream of IL-2 signaling[88]. Moreover, rapamycin treatment in humans seems not to affect the phenotype of Treg cells *in vivo*, and leads to an increase of their functionality[89]. These findings have prompted research into the use of combining IL-2 and rapamycin therapies. In NOD mice, IL-2 synergizes with the therapeutic effects of sirolimus on T1 D development, leading to a reduction in disease incidence of about 80%. The effect was further confirmed to improve islet graft survival in diabetic mice[90], although the cellular mechanisms underlying this protection have yet to be examined. In humans, exposing CD4^+ ^T cells to both IL-2 and rapamycin *in vitro *leads to an increase in the cellular frequency of FOXP3^+ ^T cells, originating from nTreg cells and *de novo *induced Treg cells[91]. Clinical trials are currently underway to assess the effects and benefits of this double therapy.

#### Combination therapy with cellular infusion

The idea of cellular therapy has also been examined. The major challenge in this case is the very low abundance of Treg cells. The possibility of expanding and/or differentiating Treg cells *in vitro *prior to re-infusing them into patients is currently the focus of several clinical trials. One major limitation to such therapy could be the lack of stability if these "artificial" Treg cells. Indeed, FOXP3^+ ^Treg cells have been shown to fluctuate in their phenotype, function, and FOXP3 expression levels upon introduction in various murine models. Subsequently, studies have highlighted the instability and heterogeneity of the Treg transcriptional signature. Hence, the risk of loss of function of massively injected Treg population, and their subsequent likely conversion into pathogenic T cells, casts doubts over the future of Treg immunotherapy. Interestingly, IL-2 has been shown to play a major role in the stabilization of the FOXP3^+ ^Treg phenotype and function[53]. Hence, IL-2 therapy could, in combination with Treg infusion, represent a plausible alternative. Indeed, low dose IL-2 in addition to donor CD4^+ ^T cell infusion has shown to significantly improve medical outcome in HSCT by increasing Treg expansion *in vivo*[92].

Alternatively, administration of selective demethylation agents and histone protein deacetylases could be considered in order to enhance Treg cell stability, as it has been shown that Foxp3 expression is modulated by DNA methylation via CpG islands in its promoter[93]. Also, as suggested by Blazar *et al*., it could be possible to use clinical-grade lentiviral vectors in order to redirect polyclonal Treg cells to the specific targets, as well as to prevent Treg cell conversion to the Teff cells[94]. Thus, Treg cells could be engineered to constantly express Foxp3, so that the infused Treg cells keep Foxp3 expression.

#### Antigen-specific immunotherapy

The efficiency of Treg-mediated immunotherapy could be greatly enhanced by focusing on auto-antigen specific Treg cells. While Treg cells can suppress antigen non-specifically *in vitro*, these cells need to home to and suppress antigen-specific responses in the target organ in order to mediate disease protection[69]. This would also reduce potential adverse effects of systemic immunosuppression in treated individuals. However, the identification and isolation of antigen-specific Treg cells, existing at very low frequencies in blood, poses significant hurdles for their use in cellular infusion protocols. A potentially promising avenue might therefore be to increase the endogenous antigen-specific Treg population. Expansion and/or *de novo *induction of Treg cells of a given specificity can theoretically be achieved by an antigen vaccination strategy. This has proven efficient in the NOD mouse model, as well as in other murine models of T1D[95-100]. The feasibility of translating these therapies to humans remains to be assessed. One potential limitation of the process is the identification of those antigens that are the most relevant as targets, as the human auto-antigen-specific T cell repertoire is diverse and the optimal antigen target could vary between patients[95]. Moreover, the possibility of conversion of antigen-specific Treg cells into Teff cells would pose an even greater danger in the context of antigen-specific Treg cell therapies. A deeper understanding of the factors that modulate this phenotypic and functional plasticity in Foxp3^+ ^Treg cells will be needed in order to implement Treg-cell based therapies in autoimmune disease.

## Conclusion

In conclusion, T1 D progression is associated with a temporal loss of CD4^+^Foxp3^+ ^Treg cells in β-islets, which perturbs the Treg/Teff cell balance and unleashes the anti-islet immune responses. Moreover, IL-2 deficiency is an important trigger to intra-islet Treg cell dysfunction and progressive loss of self-tolerance in the islets. Currently there is great interest in the use of various immunotherapeutic agents including IL-2 modulatory strategies, to prevent T1 D in genetically susceptible individuals and/or cure the overt disease. The induction and maintenance of long lasting tolerance to islet autoantigens remains a major goal of T1 D research. CD4^+ ^Treg cells represent major players in the control of T1 D and offer much hope for effective antigen-specific immunoregulation in the immediate future. However, several critical issues arise when considering the treatment of autoimmune disorders like T1D:

### - Genetic-based identification of immune defects and biomarkers of disease progression

Studies documenting quantitative or qualitative defects in CD4^+^Foxp3^+ ^Treg cells as a contributor to human T1 D are inconclusive at best. The inability to detect immune dysregulation in human T1 D as unequivocally as in the murine models could be attributed to the lack of specific and stable markers of human FOXP3^+ ^Treg cells. Indeed, the accurate immune monitoring of human Treg cell frequency and function in various clinical settings is primordial to our understanding of the fundamental role of these cells in the pathophysiology of many human diseases. Moreover, we have no reason to assume that the primary immune dysfunction is identical among individuals. Indeed, the existence of the two rodent models of the NOD mouse and the BB rat, which display distinct immune dysfunction genotypes/phenotypes, clearly demonstrates the existence of at least two distinct mechanisms that can lead to loss of γ-cell tolerance. Based on the genetic diversity of the human population, the primary dysfunction can thus be assumed to differ between individual T1 D subjects. Additionally, assuming that a primary Treg defect is important in human T1 D, it can be expected that many healthy controls will have the same defect but not get T1 D because of other genetic or environmental contributors. Conversely, this defect may not be an absolute requirement and may be absent from many of the cases. A more refined approach, based on genetic-based selection of clinically stratified T1 D subjects, may now be feasible, given the recent breakthroughs in the genetics of T1D[101]. Knowledge of how known and novel T1 D loci affect Treg cell development and function can be expected to lead to assessments of immune function that provide meaningful information for the individual being tested.

The detection of T1D-specific antibodies is currently used for meaningful and reliable prediction of T1 D, years before clinical onset, but likely reflects ongoing autoimmune responses towards β-islets. Although still under development, assays of immune responses, and in particular antigen-specific T cell responses could become an alternative screening tool. However assays are urgently required to measure not only the number/function in pro-inflammatory, diabetogenic cells, but also the induction, expansion and function of islet-specific Treg cells. Reliable assays to detect a primary (i.e. genetically determined and preceding the autoimmune process) immune dysfunction exist in the rodent models but not in humans. Hence the critical question remains of whether biomarkers can be developed to detect the primary, genetically-determined, immune dysfunction that leads to T1 D rather than the consequences of autoimmunity induced on a given genetic background by environmental triggers.

### - When could a treatment be initiated/applied universally to all T1D-susceptible subjects?

T1 D develops progressively, over several years, and is only diagnosed once most of the damage to the pancreas has already been done. Insights into human pathogenesis are scarce, but the NOD model displays a step-wise pathogenesis, whereby insulitis occurs long before islet-destruction. This suggests the existence of several so-called checkpoints, when distinct immunological events are at play. As such, therapeutic intervention can be expected to have a different impact, depending on what stage the disease development is at. These pathogenesis phases, however, are still ill-defined in humans. The genetic and physiological hallmarks of disease risk and progression have previously been thoroughly reviewed[101].

## Competing interests

The authors declare that they have no competing interests.

## Authors' contributions

All authors contributed to the writing of this manuscript. All authors have read and approved the final manuscript.

## References

[B1] Piccirillo CA d'Hennezel E Sgouroudis E Yurchenko E CD4+Foxp3+ regulatory T cells in the control of autoimmunity: in vivo veritas Curr Opin Immunol 2008 20 655 662 10.1016/j.coi.2008.09.006 18926906

[B2] Hori S Nomura T Sakaguchi S Control of regulatory T cell development by the transcription factor Foxp3 Science 2003 299 1057 1061 10.1126/science.1079490 28115586

[B3] Fontenot JD Rasmussen JP Gavin MA Rudensky AY A function for interleukin 2 in Foxp3-expressing regulatory T cells Nat Immunol 2005 6 1142 1151 10.1038/ni1263 16227984

[B4] d'Hennezel E Ben-Shoshan M Ochs HD Torgerson TR Russell LJ Lejtenyi C Noya FJ Jabado N Mazer B Piccirillo CA FOXP3 forkhead domain mutation and regulatory T cells in the IPEX syndrome N Engl J Med 2009 361 1710 1713 10.1056/NEJMc0907093 19846862

[B5] Khattri R Cox T Yasayko SA Ramsdell F An essential role for Scurfin in CD4+CD25+ T regulatory cells Nat Immunol 2003 4 337 342 10.1038/ni909 12612581

[B6] Bach JF Chatenoud L Tolerance to islet autoantigens in type 1 diabetes Annu Rev Immunol 2001 19 131 161 10.1146/annurev.immunol.19.1.131 11244033

[B7] Anderson MS Bluestone JA The NOD mouse: a model of immune dysregulation AnnuRevImmunol 2005 23 447 485 10.1146/annurev.immunol.23.021704.115643 15771578

[B8] Tritt M Sgouroudis E d'Hennezel E Albanese A Piccirillo CA Functional waning of naturally occurring CD4+ regulatory T-cells contributes to the onset of autoimmune diabetes Diabetes 2008 57 113 123 10.2337/db06-1700 17928397

[B9] Sgouroudis E Albanese A Piccirillo CA Impact of protective IL-2 allelic variants on CD4+ Foxp3+ regulatory T cell function in situ and resistance to autoimmune diabetes in NOD mice J Immunol 2008 181 6283 6292 18941219 10.4049/jimmunol.181.9.6283

[B10] Tang Q Adams JY Penaranda C Melli K Piaggio E Sgouroudis E Piccirillo CA Salomon BL Bluestone JA Central role of defective interleukin-2 production in the triggering of islet autoimmune destruction Immunity 2008 28 687 697 10.1016/j.immuni.2008.03.016 18468463 PMC2394854

[B11] Salomon B Lenschow DJ Rhee L Ashourian N Singh B Sharpe A Bluestone JA B7/CD28 costimulation is essential for the homeostasis of the CD4+CD25+ immunoregulatory T cells that control autoimmune diabetes Immunity 2000 12 431 440 10.1016/S1074-7613(00)80195-8 10795741

[B12] Tang Q Henriksen KJ Boden EK Tooley AJ Ye J Subudhi SK Zheng XX Strom TB Bluestone JA Cutting edge: CD28 controls peripheral homeostasis of CD4+CD25+ regulatory T cells J Immunol 2003 171 3348 3352 14500627 10.4049/jimmunol.171.7.3348

[B13] Kumanogoh A Wang X Lee I Watanabe C Kamanaka M Shi W Yoshida K Sato T Habu S Itoh M Increased T cell autoreactivity in the absence of CD40-CD40 ligand interactions: a role of CD40 in regulatory T cell development J Immunol 2001 166 353 360 11123312 10.4049/jimmunol.166.1.353

[B14] Nelson BH Willerford DM Biology of the interleukin-2 receptor Adv Immunol 1998 70 1 81 full_text 9755337 10.1016/s0065-2776(08)60386-7

[B15] Malek TR The biology of interleukin-2 Annu Rev Immunol 2008 26 453 479 10.1146/annurev.immunol.26.021607.090357 18062768

[B16] Wolf M Schimpl A Hunig T Control of T cell hyperactivation in IL-2-deficient mice by CD4(+)CD25(-) and CD4(+)CD25(+) T cells: evidence for two distinct regulatory mechanisms Eur J Immunol 2001 31 1637 1645 10.1002/1521-4141(200106)31:6<1637::AID-IMMU1637>3.0.CO;2-T 11385607

[B17] Caudy AA Reddy ST Chatila T Atkinson JP Verbsky JW CD25 deficiency causes an immune dysregulation, polyendocrinopathy, enteropathy, X-linked-like syndrome, and defective IL-10 expression from CD4 lymphocytes J Allergy Clin Immunol 2007 119 482 487 10.1016/j.jaci.2006.10.007 17196245

[B18] Roifman CM Human IL-2 receptor alpha chain deficiency Pediatr Res 2000 48 6 11 10.1203/00006450-200007000-00004 10879793

[B19] Bernasconi A Marino R Ribas A Rossi J Ciaccio M Oleastro M Ornani A Paz R Rivarola MA Zelazko M Belgorosky A Characterization of immunodeficiency in a patient with growth hormone insensitivity secondary to a novel STAT5b gene mutation Pediatrics 2006 118 e1584 1592 10.1542/peds.2005-2882 17030597

[B20] Lyons PA Armitage N Argentina F Denny P Hill NJ Lord CJ Wilusz MB Peterson LB Wicker LS Todd JA Congenic mapping of the type 1 diabetes locus, Idd3, to a 780-kb region of mouse chromosome 3: identification of a candidate segment of ancestral DNA by haplotype mapping Genome Res 2000 10 446 453 10.1101/gr.10.4.446 10779485 PMC310860

[B21] Denny P Lord CJ Hill NJ Goy JV Levy ER Podolin PL Peterson LB Wicker LS Todd JA Lyons PA Mapping of the IDDM locus Idd3 to a 0.35-cM interval containing the interleukin-2 gene Diabetes 1997 46 695 700 10.2337/diabetes.46.4.695 9075813

[B22] Yamanouchi J Rainbow D Serra P Howlett S Hunter K Garner VE Gonzalez-Munoz A Clark J Veijola R Cubbon R Interleukin-2 gene variation impairs regulatory T cell function and causes autoimmunity Nat Genet 2007 39 329 337 10.1038/ng1958 17277778 PMC2886969

[B23] Encinas JA Kuchroo VK Genetics of experimental autoimmune encephalomyelitis Curr Dir Autoimmun 1999 1 247 272 full_text 11791445 10.1159/000060485

[B24] Podolin PL Wilusz MB Cubbon RM Pajvani U Lord CJ Todd JA Peterson LB Wicker LS Lyons PA Differential glycosylation of interleukin 2, the molecular basis for the NOD Idd3 type 1 diabetes gene? Cytokine 2000 12 477 482 10.1006/cyto.1999.0609 10857762

[B25] van Heel DA Franke L Hunt KA Gwilliam R Zhernakova A Inouye M Wapenaar MC Barnardo MC Bethel G Holmes GK A genome-wide association study for celiac disease identifies risk variants in the region harboring IL2 and IL21 Nat Genet 2007 39 827 829 10.1038/ng2058 17558408 PMC2274985

[B26] Todd JA Walker NM Cooper JD Smyth DJ Downes K Plagnol V Bailey R Nejentsev S Field SF Payne F Robust associations of four new chromosome regions from genome-wide analyses of type 1 diabetes Nat Genet 2007 39 857 864 10.1038/ng2068 17554260 PMC2492393

[B27] Zhernakova A Alizadeh BZ Bevova M van Leeuwen MA Coenen MJ Franke B Franke L Posthumus MD van Heel DA van der Steege G Novel association in chromosome 4q27 region with rheumatoid arthritis and confirmation of type 1 diabetes point to a general risk locus for autoimmune diseases Am J Hum Genet 2007 81 1284 1288 10.1086/522037 17999365 PMC2276343

[B28] Vella A Cooper JD Lowe CE Walker N Nutland S Widmer B Jones R Ring SM McArdle W Pembrey ME Localization of a type 1 diabetes locus in the IL2RA/CD25 region by use of tag single-nucleotide polymorphisms Am J Hum Genet 2005 76 773 779 10.1086/429843 15776395 PMC1199367

[B29] Qu HQ Montpetit A Ge B Hudson TJ Polychronakos C Toward further mapping of the association between the IL2RA locus and type 1 diabetes Diabetes 2007 56 1174 1176 10.2337/db06-1555 17395754

[B30] Qu HQ Bradfield JP Belisle A Grant SF Hakonarson H Polychronakos C The type I diabetes association of the IL2RA locus Genes Immun 2009 10 Suppl 1 S42 48 10.1038/gene.2009.90 19956099 PMC2805446

[B31] Lowe CE Cooper JD Brusko T Walker NM Smyth DJ Bailey R Bourget K Plagnol V Field S Atkinson M Large-scale genetic fine mapping and genotype-phenotype associations implicate polymorphism in the IL2RA region in type 1 diabetes Nat Genet 2007 39 1074 1082 10.1038/ng2102 17676041

[B32] Maier LM Lowe CE Cooper J Downes K Anderson DE Severson C Clark PM Healy B Walker N Aubin C IL2RA genetic heterogeneity in multiple sclerosis and type 1 diabetes susceptibility and soluble interleukin-2 receptor production PLoS Genet 2009 5 e1000322 10.1371/journal.pgen.1000322 19119414 PMC2602853

[B33] Qu HQ Verlaan DJ Ge B Lu Y Lam KC Grabs R Harmsen E Hudson TJ Hakonarson H Pastinen T Polychronakos C A cis-acting regulatory variant in the IL2RA locus J Immunol 2009 183 5158 5162 10.4049/jimmunol.0901337 19794070

[B34] Dendrou CA Plagnol V Fung E Yang JH Downes K Cooper JD Nutland S Coleman G Himsworth M Hardy M Cell-specific protein phenotypes for the autoimmune locus IL2RA using a genotype-selectable human bioresource Nat Genet 2009 41 1011 1015 10.1038/ng.434 19701192 PMC2749506

[B35] Klinker MW Schiller JJ Magnuson VL Wang T Basken J Veth K Pearce KI Kinnunen L Harjutsalo V Wang X Single-nucleotide polymorphisms in the IL2RA gene are associated with age at diagnosis in late-onset Finnish type 1 diabetes subjects Immunogenetics 2010 62 101 107 10.1007/s00251-009-0417-4 20033399

[B36] Kawasaki E Awata T Ikegami H Kobayashi T Maruyama T Nakanishi K Shimada A Uga M Kurihara S Kawabata Y Genetic association between the interleukin-2 receptor-alpha gene and mode of onset of type 1 diabetes in the Japanese population J Clin Endocrinol Metab 2009 94 947 952 10.1210/jc.2008-1596 19106270

[B37] Pop SM Wong CP Culton DA Clarke SH Tisch R Single cell analysis shows decreasing FoxP3 and TGFbeta1 coexpressing CD4+CD25+ regulatory T cells during autoimmune diabetes J Exp Med 2005 201 1333 1346 10.1084/jem.20042398 15837817 PMC2213147

[B38] Brusko TM Wasserfall CH Clare-Salzler MJ Schatz DA Atkinson MA Functional defects and the influence of age on the frequency of CD4+ CD25+ T-cells in type 1 diabetes Diabetes 2005 54 1407 1414 10.2337/diabetes.54.5.1407 15855327

[B39] Chen Z Herman AE Matos M Mathis D Benoist C Where CD4+CD25+ T reg cells impinge on autoimmune diabetes J Exp Med 2005 202 1387 1397 10.1084/jem.20051409 16301745 PMC2212985

[B40] Feuerer M Hill JA Mathis D Benoist C Foxp3+ regulatory T cells: differentiation, specification, subphenotypes Nat Immunol 2009 10 689 695 10.1038/ni.1760 19536194

[B41] D'Alise AM Auyeung V Feuerer M Nishio J Fontenot J Benoist C Mathis D The defect in T-cell regulation in NOD mice is an effect on the T-cell effectors Proc Natl Acad Sci USA 2008 105 19857 19862 10.1073/pnas.0810713105 19073938 PMC2604930

[B42] You S Belghith M Cobbold S Alyanakian MA Gouarin C Barriot S Garcia C Waldmann H Bach JF Chatenoud L Autoimmune diabetes onset results from qualitative rather than quantitative age-dependent changes in pathogenic T-cells Diabetes 2005 54 1415 1422 10.2337/diabetes.54.5.1415 15855328

[B43] D'Cruz LM Klein L Development and function of agonist-induced CD25+Foxp3+ regulatory T cells in the absence of interleukin 2 signaling Nat Immunol 2005 6 1152 1159 10.1038/ni1264 16227983

[B44] Malek TR Porter BO Codias EK Scibelli P Yu A Normal lymphoid homeostasis and lack of lethal autoimmunity in mice containing mature T cells with severely impaired IL-2 receptors J Immunol 2000 164 2905 2914 10706676 10.4049/jimmunol.164.6.2905

[B45] Malek TR Yu A Vincek V Scibelli P Kong L CD4 regulatory T cells prevent lethal autoimmunity in IL-2Rbeta-deficient mice. Implications for the nonredundant function of IL-2 Immunity 2002 17 167 178 10.1016/S1074-7613(02)00367-9 12196288

[B46] Setoguchi R Hori S Takahashi T Sakaguchi S Homeostatic maintenance of natural Foxp3(+) CD25(+) CD4(+) regulatory T cells by interleukin (IL)-2 and induction of autoimmune disease by IL-2 neutralization J Exp Med 2005 201 723 735 10.1084/jem.20041982 15753206 PMC2212841

[B47] Burchill MA Goetz CA Prlic M O'Neil JJ Harmon IR Bensinger SJ Turka LA Brennan P Jameson SC Farrar MA Distinct effects of STAT5 activation on CD4+ and CD8+ T cell homeostasis: development of CD4+CD25+ regulatory T cells versus CD8+ memory T cells J Immunol 2003 171 5853 5864 14634095 10.4049/jimmunol.171.11.5853

[B48] Antov A Yang L Vig M Baltimore D Van Parijs L Essential role for STAT5 signaling in CD25+CD4+ regulatory T cell homeostasis and the maintenance of self-tolerance J Immunol 2003 171 3435 3441 14500638 10.4049/jimmunol.171.7.3435

[B49] Setoguchi R Hori S Takahashi T Sakaguchi S Homeostatic maintenance of natural Foxp3(+) CD25(+) CD4(+) regulatory T cells by interleukin (IL)-2 and induction of autoimmune disease by IL-2 neutralization J Exp Med 2005 201 723 735 10.1084/jem.20041982 15753206 PMC2212841

[B50] Elliott EA Flavell RA Transgenic mice expressing constitutive levels of IL-2 in islet beta cells develop diabetes Int Immunol 1994 6 1629 1637 10.1093/intimm/6.11.1629 7865456

[B51] Sgouroudis E Piccirillo CA Control of type 1 diabetes by CD4+Foxp3+ regulatory T cells: lessons from mouse models and implications for human disease Diabetes Metab Res Rev 2009 25 208 218 10.1002/dmrr.945 19214972

[B52] Zheng SG Wang J Wang P Gray JD Horwitz DA IL-2 Is Essential for TGF-beta to Convert Naive CD4+CD25- Cells to CD25+Foxp3+ Regulatory T Cells and for Expansion of These Cells J Immunol 2007 178 2018 2027 17277105 10.4049/jimmunol.178.4.2018

[B53] Zhou X Bailey-Bucktrout S Jeker LT Bluestone JA Plasticity of CD4(+) FoxP3(+) T cells Curr Opin Immunol 2009 21 281 285 10.1016/j.coi.2009.05.007 19500966 PMC2733784

[B54] Komatsu N Mariotti-Ferrandiz ME Wang Y Malissen B Waldmann H Hori S Heterogeneity of natural Foxp3+ T cells: a committed regulatory T-cell lineage and an uncommitted minor population retaining plasticity Proc Natl Acad Sci USA 2009 106 1903 1908 10.1073/pnas.0811556106 19174509 PMC2644136

[B55] Lu LF Thai TH Calado DP Chaudhry A Kubo M Tanaka K Loeb GB Lee H Yoshimura A Rajewsky K Rudensky AY Foxp3-dependent microRNA155 confers competitive fitness to regulatory T cells by targeting SOCS1 protein Immunity 2009 30 80 91 10.1016/j.immuni.2008.11.010 19144316 PMC2654249

[B56] Jeker J Bluestone JA Small RNA regulators of T cell-mediated autoimmunity J Clin Immunol 2010 30 347 357 10.1007/s10875-010-9392-7 20393792

[B57] Pauley KM Cha S Chan EK MicroRNA in autoimmunity and autoimmune diseases J Autoimmun 2009 32 189 194 10.1016/j.jaut.2009.02.012 19303254 PMC2717629

[B58] Zhou X Jeker LT Fife BT Zhu S Anderson MS McManus MT Bluestone JA Selective miRNA disruption in T reg cells leads to uncontrolled autoimmunity J Exp Med 2008 205 1983 1991 10.1084/jem.20080707 18725525 PMC2526194

[B59] Kukreja A Cost G Marker J Zhang C Sun Z Lin-Su K Ten S Sanz M Exley M Wilson B Multiple immuno-regulatory defects in type-1 diabetes J Clin Invest 2002 109 131 140 11781358 10.1172/JCI13605 PMC150819

[B60] Lindley S Dayan CM Bishop A Roep BO Peakman M Tree TI Defective suppressor function in CD4(+)CD25(+) T-cells from patients with type 1 diabetes Diabetes 2005 54 92 99 10.2337/diabetes.54.1.92 15616015

[B61] Brusko T Wasserfall C McGrail K Schatz R Viener HL Schatz D Haller M Rockell J Gottlieb P Clare-Salzler M Atkinson M No alterations in the frequency of FOXP3+ regulatory T-cells in type 1 diabetes Diabetes 2007 56 604 612 10.2337/db06-1248 17327427

[B62] Putnam AL Vendrame F Dotta F Gottlieb PA CD4+CD25high regulatory T cells in human autoimmune diabetes J Autoimmun 2005 24 55 62 10.1016/j.jaut.2004.11.004 15725577

[B63] Dendrou CA Wicker LS The IL-2/CD25 pathway determines susceptibility to T1 D in humans and NOD mice J Clin Immunol 2008 28 685 696 10.1007/s10875-008-9237-9 18780166

[B64] Allan SE Passerini L Bacchetta R Crellin N Dai M Orban PC Ziegler SF Roncarolo MG Levings MK The role of 2 FOXP3 isoforms in the generation of human CD4+ Tregs J Clin Invest 2005 115 3276 3284 10.1172/JCI24685 16211090 PMC1242190

[B65] Passerini L Allan SE Battaglia M Di Nunzio S Alstad AN Levings MK Roncarolo MG Bacchetta R STAT5-signaling cytokines regulate the expression of FOXP3 in CD4+CD25+ regulatory T cells and CD4+CD25- effector T cells Int Immunol 2008 20 421 431 10.1093/intimm/dxn002 18270368

[B66] Zorn E Nelson EA Mohseni M Porcheray F Kim H Litsa D Bellucci R Raderschall E Canning C Soiffer RJ IL-2 regulates FOXP3 expression in human CD4+CD25+ regulatory T cells through a STAT-dependent mechanism and induces the expansion of these cells in vivo Blood 2006 108 1571 1579 10.1182/blood-2006-02-004747 16645171 PMC1895505

[B67] Long SA Cerosaletti K Bollyky PL Tatum M Shilling H Zhang S Zhang ZY Pihoker C Sanda S Greenbaum C Buckner JH Defects in IL-2R signaling contribute to diminished maintenance of FOXP3 expression in CD4(+)CD25(+) regulatory T-cells of type 1 diabetic subjects Diabetes 2010 59 407 415 10.2337/db09-0694 19875613 PMC2809970

[B68] Jailwala P Waukau J Glisic S Jana S Ehlenbach S Hessner M Alemzadeh R Matsuyama S Laud P Wang X Ghosh S Apoptosis of CD4+ CD25(high) T cells in type 1 diabetes may be partially mediated by IL-2 deprivation PLoS One 2009 4 e6527 10.1371/journal.pone.0006527 19654878 PMC2716541

[B69] Lennon GP Bettini M Burton AR Vincent E Arnold PY Santamaria P Vignali DA T cell islet accumulation in type 1 diabetes is a tightly regulated, cell-autonomous event Immunity 2009 31 643 653 10.1016/j.immuni.2009.07.008 19818656 PMC2763021

[B70] Trudeau JD Kelly-Smith C Verchere CB Elliott JF Dutz JP Finegood DT Santamaria P Tan R Prediction of spontaneous autoimmune diabetes in NOD mice by quantification of autoreactive T cells in peripheral blood J Clin Invest 2003 111 217 223 12531877 10.1172/JCI16409 PMC151866

[B71] Eisenbarth GS Kotzin BL Enumerating autoreactive T cells in peripheral blood: a big step in diabetes prediction The Journal of Clinical Investigation 2003 111 179 181 12531872 10.1172/JCI17621 PMC151887

[B72] Wong CP Stevens R Long B Li L Wang Y Wallet MA Goudy KS Frelinger JA Tisch R Identical beta cell-specific CD8(+) T cell clonotypes typically reside in both peripheral blood lymphocyte and pancreatic islets J Immunol 2007 178 1388 1395 17237386 10.4049/jimmunol.178.3.1388

[B73] Pang S Zhang L Wang H Yi Z Li L Gao L Zhao J Tisch R Katz JD Wang B CD8(+) T cells specific for beta cells encounter their cognate antigens in the islets of NOD mice Eur J Immunol 2009 39 2716 2724 10.1002/eji.200939408 19658094

[B74] Li L He Q Garland A Yi Z Aybar LT Kepler TB Frelinger JA Wang B Tisch R beta cell-specific CD4+ T cell clonotypes in peripheral blood and the pancreatic islets are distinct J Immunol 2009 183 7585 7591 10.4049/jimmunol.0901587 19917704

[B75] Oling V Marttila J Ilonen J Kwok WW Nepom G Knip M Simell O Reijonen H GAD65- and proinsulin-specific CD4+ T-cells detected by MHC class II tetramers in peripheral blood of type 1 diabetes patients and at-risk subjects J Autoimmun 2005 25 235 243 10.1016/j.jaut.2005.09.018 16263242

[B76] Reijonen H Novak EJ Kochik S Heninger A Liu AW Kwok WW Nepom GT Detection of GAD65-specific T-cells by major histocompatibility complex class II tetramers in type 1 diabetic patients and at-risk subjects Diabetes 2002 51 1375 1382 10.2337/diabetes.51.5.1375 11978633

[B77] Danke NA Yang J Greenbaum C Kwok WW Comparative study of GAD65-specific CD4+ T cells in healthy and type 1 diabetic subjects J Autoimmun 2005 25 303 311 10.1016/j.jaut.2005.08.007 16249070

[B78] Monti P Scirpoli M Rigamonti A Mayr A Jaeger A Bonfanti R Chiumello G Ziegler AG Bonifacio E Evidence for in vivo primed and expanded autoreactive T cells as a specific feature of patients with type 1 diabetes J Immunol 2007 179 5785 5792 17947651 10.4049/jimmunol.179.9.5785

[B79] Ahmadzadeh M Rosenberg SA IL-2 administration increases CD4+ CD25(hi) Foxp3+ regulatory T cells in cancer patients Blood 2006 107 2409 2414 10.1182/blood-2005-06-2399 16304057 PMC1473973

[B80] Yu A Zhu L Altman NH Malek TR A low interleukin-2 receptor signaling threshold supports the development and homeostasis of T regulatory cells Immunity 2009 30 204 217 10.1016/j.immuni.2008.11.014 19185518 PMC2962446

[B81] Slingluff CL Jr Petroni GR Yamshchikov GV Hibbitts S Grosh WW Chianese-Bullock KA Bissonette EA Barnd DL Deacon DH Patterson JW Immunologic and clinical outcomes of vaccination with a multiepitope melanoma peptide vaccine plus low-dose interleukin-2 administered either concurrently or on a delayed schedule J Clin Oncol 2004 22 4474 4485 10.1200/JCO.2004.10.212 15542798

[B82] Boyman O Surh CD Sprent J Potential use of IL-2/anti-IL-2 antibody immune complexes for the treatment of cancer and autoimmune disease Expert Opin Biol Ther 2006 6 1323 1331 10.1517/14712598.6.12.1323 17223740

[B83] Boyman O Kovar M Rubinstein MP Surh CD Sprent J Selective stimulation of T cell subsets with antibody-cytokine immune complexes Science 2006 311 1924 1927 10.1126/science.1122927 16484453

[B84] Kamimura D Bevan MJ Naive CD8+ T cells differentiate into protective memory-like cells after IL-2 anti IL-2 complex treatment in vivo J Exp Med 2007 204 1803 1812 10.1084/jem.20070543 17664293 PMC2118678

[B85] Letourneau S van Leeuwen EM Krieg C Martin C Pantaleo G Sprent J Surh CD Boyman O IL-2/anti-IL-2 antibody complexes show strong biological activity by avoiding interaction with IL-2 receptor alpha subunit CD25 Proc Natl Acad Sci USA 2010 107 2171 2176 10.1073/pnas.0909384107 20133862 PMC2836659

[B86] Mondino A Mueller DL mTOR at the crossroads of T cell proliferation and tolerance Semin Immunol 2007 19 162 172 10.1016/j.smim.2007.02.008 17383196 PMC1995654

[B87] Strauss L Czystowska M Szajnik M Mandapathil M Whiteside TL Differential responses of human regulatory T cells (Treg) and effector T cells to rapamycin PLoS One 2009 4 e5994 10.1371/journal.pone.0005994 19543393 PMC2694984

[B88] Zeiser R Negrin RS Interleukin-2 receptor downstream events in regulatory T cells: implications for the choice of immunosuppressive drug therapy Cell Cycle 2008 7 458 462 10.4161/cc.7.4.5454 18235249 PMC2886808

[B89] Monti P Scirpoli M Maffi P Piemonti L Secchi A Bonifacio E Roncarolo MG Battaglia M Rapamycin monotherapy in patients with type 1 diabetes modifies CD4+CD25+FOXP3+ regulatory T-cells Diabetes 2008 57 2341 2347 10.2337/db08-0138 18559659 PMC2518485

[B90] Rabinovitch A Suarez-Pinzon WL Shapiro AM Rajotte RV Power R Combination therapy with sirolimus and interleukin-2 prevents spontaneous and recurrent autoimmune diabetes in NOD mice Diabetes 2002 51 638 645 10.2337/diabetes.51.3.638 11872661

[B91] Long SA Buckner JH Combination of rapamycin and IL-2 increases de novo induction of human CD4(+)CD25(+)FOXP3(+) T cells J Autoimmun 2008 30 293 302 10.1016/j.jaut.2007.12.012 18313267 PMC2431984

[B92] Zorn E Mohseni M Kim H Porcheray F Lynch A Bellucci R Canning C Alyea EP Soiffer RJ Ritz J Combined CD4+ donor lymphocyte infusion and low-dose recombinant IL-2 expand FOXP3+ regulatory T cells following allogeneic hematopoietic stem cell transplantation Biol Blood Marrow Transplant 2009 15 382 388 10.1016/j.bbmt.2008.12.494 19203731 PMC2678797

[B93] Kim JM Rasmussen JP Rudensky AY Regulatory T cells prevent catastrophic autoimmunity throughout the lifespan of mice Nat Immunol 2007 8 191 197 10.1038/ni1428 17136045

[B94] Riley JL June CH Blazar BR Human T regulatory cell therapy: take a billion or so and call me in the morning Immunity 2009 30 656 665 10.1016/j.immuni.2009.04.006 19464988 PMC2742482

[B95] Wang B Tisch R Parameters influencing antigen-specific immunotherapy for Type 1 diabetes Immunologic Research 2008 42 246 258 10.1007/s12026-008-8090-5 19052699

[B96] Casares S Hurtado A McEvoy RC Sarukhan A von Boehmer H Brumeanu TD Down-regulation of diabetogenic CD4+ T cells by a soluble dimeric peptide-MHC class II chimera Nat Immunol 2002 3 383 391 10.1038/ni770 11862219

[B97] Tisch R Liblau RS Yang XD Liblau P McDevitt HO Induction of GAD65-specific regulatory T-cells inhibits ongoing autoimmune diabetes in nonobese diabetic mice Diabetes 1998 47 894 899 10.2337/diabetes.47.6.894 9604865

[B98] Fife BT Guleria I Gubbels Bupp M Eagar TN Tang Q Bour-Jordan H Yagita H Azuma M Sayegh MH Bluestone JA Insulin-induced remission in new-onset NOD mice is maintained by the PD-1-PD-L1 pathway J Exp Med 2006 203 2737 2747 10.1084/jem.20061577 17116737 PMC2118162

[B99] Jain R Tartar DM Gregg RK Divekar RD Bell JJ Lee HH Yu P Ellis JS Hoeman CM Franklin CL Zaghouani H Innocuous IFNgamma induced by adjuvant-free antigen restores normoglycemia in NOD mice through inhibition of IL-17 production J Exp Med 2008 205 207 218 10.1084/jem.20071878 18195074 PMC2234380

[B100] Homann D Holz A Bot A Coon B Wolfe T Petersen J Dyrberg TP Grusby MJ von Herrath MG Autoreactive CD4+ T cells protect from autoimmune diabetes via bystander suppression using the IL-4/Stat6 pathway Immunity 1999 11 463 472 10.1016/S1074-7613(00)80121-1 10549628

[B101] Ziegler AG Nepom GT Prediction and pathogenesis in type 1 diabetes Immunity 2010 32 468 478 10.1016/j.immuni.2010.03.018 20412757 PMC2861716

